# CD19 CAR-T cell therapy in large B-cell lymphoma: clinical evidence, resistance, toxicity, and precision strategies

**DOI:** 10.3389/fimmu.2026.1903457

**Published:** 2026-07-24

**Authors:** Congcong Wang, Jun Zhu, Jiaojiao Qiao, Huahua Wang, Xue Liang

**Affiliations:** 1 Department of Hematology, The 989th Hospital of the Joint Logistics Support Force of Chinese People's Liberation Army, Luoyang, China; 2 Department of General Surgery, Southern Theater Air Force Hospital, Guangzhou, China; 3 Department of Pharmacy, Yichuan People’s Hospital, Luoyang, Henan, China

**Keywords:** CAR T-cell therapy, CD19, clinical trials, CtDNA, diffuse large B-cell lymphoma, large B-cell lymphoma, precision immunotherapy, real-world evidence

## Abstract

CD19-directed chimeric antigen receptor (CAR) T-cell therapy has transformed the management of relapsed or refractory large B-cell lymphoma (LBCL), producing durable remissions in a subset of patients whose disease previously had few curative options. Axicabtagene ciloleucel, tisagenlecleucel, and lisocabtagene maraleucel established CAR T-cell therapy in the third-line setting, and randomized studies subsequently moved axicabtagene ciloleucel and lisocabtagene maraleucel into second-line treatment for primary refractory or early relapsed disease. This review provides a clinically anchored, mechanism-focused synthesis of CAR T-cell therapy in LBCL. We critically compare pivotal trials, long-term follow-up, patient-selection principles, and real-world evidence, emphasizing that apparent differences across products must be interpreted in light of eligibility criteria, analytic denominators, bridging therapy, manufacturing intervals, toxicity grading, and treatment crossover. We then examine resistance and relapse as systems-level phenomena arising from antigen modulation, tumor-intrinsic evolution, impaired CAR T-cell fitness, suppressive myeloid and stromal networks, systemic inflammation, metabolic stress, and incomplete immune recovery. The biological basis and clinical implications of cytokine release syndrome, immune effector cell-associated neurotoxicity syndrome, prolonged cytopenias, infections, and late nonrelapse mortality are also reviewed. Finally, we discuss circulating tumor DNA, metabolic imaging, single-cell and multi-omic profiling, artificial intelligence, dual-target and armored constructs, allogeneic platforms, and *in vivo* CAR programming as components of precision cellular therapy. The central clinical challenge is no longer whether CAR T-cell therapy can work, but how to select patients, deliver treatment rapidly, anticipate failure, and preserve long-term immune and functional health.

## 1 Introduction

Large B-cell lymphoma (LBCL) comprises a clinically aggressive and biologically heterogeneous group of mature B-cell malignancies. Although rituximab-containing anthracycline-based immunochemotherapy is curative for many patients, a substantial proportion develop primary refractory disease or relapse after frontline treatment (1). Historically, potentially curative management relied on platinum-based salvage chemotherapy followed by autologous stem cell transplantation (ASCT) in patients with chemosensitive disease (2, 3). This strategy is substantially less effective in primary refractory or early relapsed LBCL and is not feasible for many patients because of disease kinetics, comorbidity, organ dysfunction, or failure to achieve a salvage response (4).

CD19-directed CAR T-cell therapy changed this treatment paradigm by introducing a living immune product capable of antigen recognition, *in vivo* expansion, cytotoxic activity, and, in a subset of patients, durable immune surveillance (5–8). The pivotal ZUMA-1, JULIET, and TRANSCEND NHL 001 studies established activity in heavily pretreated disease (5, 7, 8), whereas ZUMA-7, TRANSFORM, and BELINDA tested whether CAR T-cell therapy could replace salvage chemotherapy and ASCT in second-line early relapse or refractory disease (9–12). These studies collectively demonstrate that efficacy depends not only on the CAR construct, but also on when treatment is delivered, whether the patient reaches infusion, how disease is controlled during manufacturing, and how outcomes are defined.

Resistance after CAR T-cell therapy is best understood as a systems-level problem (13–18). CD19 loss or downregulation explains only a subset of relapses, and many failures occur despite preserved target expression (16, 17). Impaired CAR T-cell fitness, chronic antigen-driven exhaustion, suppressive myeloid circuits, hostile metabolic conditions, systemic inflammation, and delayed immune reconstitution frequently coexist (13–18). Toxicity is similarly multidimensional: cytokine release syndrome (CRS) and immune effector cell-associated neurotoxicity syndrome (ICANS) dominate early management, whereas prolonged cytopenias, infection, hypogammaglobulinemia, and late nonrelapse mortality increasingly define survivorship (19–26).

## 2 Clinical evolution of CAR-T therapy in large B-cell lymphoma

### 2.1 From late-line salvage therapy to earlier-line intervention

The pivotal third-line studies established that CD19 CAR T-cell therapy could induce complete remission in chemotherapy-refractory LBCL, but they differed materially in eligibility, manufacturing, bridging therapy, toxicity grading, and analytic denominator. In ZUMA-1, axicabtagene ciloleucel (axi-cel) produced an objective response rate (ORR) of 83% and a complete response (CR) rate of 58% among treated patients (5, 27). JULIET reported an ORR of approximately 53% and a CR rate of 39% with tisagenlecleucel (tisa-cel) (7, 28), whereas TRANSCEND NHL 001 reported an ORR of 73% and a CR rate of 53% with lisocabtagene maraleucel (liso-cel) (8, 29). These results were unprecedented in a population with historically poor outcomes, but the numerical differences should not be interpreted as head-to-head product superiority.

Operational design is part of the therapeutic effect. ZUMA-1 did not permit protocol-defined cytotoxic bridging chemotherapy, thereby selecting patients able to remain clinically stable through a relatively rapid treatment pathway. JULIET permitted bridging therapy and had a longer, globally distributed manufacturing pathway, making attrition between enrollment and infusion more clinically relevant. TRANSCEND used a defined CD4-positive/CD8-positive composition and included a comparatively broad clinical population. Consequently, infused-patient response rates, intention-to-treat feasibility, and the probability of reaching infusion answer different questions. A clinically useful synthesis must distinguish biological efficacy after infusion from the effectiveness of the entire referral-to-infusion pathway.

Long-term follow-up supports a plateau in failure risk for a subset of patients. At 5 years in ZUMA-1, progression-free survival (PFS) and overall survival (OS) were approximately 32% and 43%, respectively; among patients achieving CR, the estimated 5-year OS was approximately 64% (27). In the 5-year JULIET analysis, PFS and OS were approximately 28% and 32% for all infused patients, and the 60-month relapse-free probability was 61% among responders (28). In TRANSCEND, median duration of response, PFS, and OS at extended follow-up were 23.1, 6.8, and 27.3 months, respectively (29). These data support curative potential, but they also show that early nonresponse and early relapse remain the dominant efficacy gap.

### 2.2 Randomized second-line trials: why ZUMA-7, TRANSFORM, and BELINDA diverged

Randomized second-line trials addressed patients with primary refractory disease or relapse within 12 months after frontline therapy, a group with limited benefit from conventional salvage chemotherapy followed by ASCT (9–12). ZUMA-7 demonstrated superior event-free survival (EFS; 8.3 vs 2.0 months) and CR rate (65% vs 32%) with axi-cel compared with standard care (9). With 47.2 months of median follow-up, the 4-year OS was 54.6% with axi-cel and 46.0% with standard care (hazard ratio for death, 0.73), despite frequent post-progression cellular therapy in the control arm (10). These results establish that earlier cellular therapy can improve survival rather than merely increase response rate.

TRANSFORM similarly favored liso-cel over salvage chemotherapy and ASCT (11). In the 3-year analysis, median EFS was 29.5 months with liso-cel versus 2.4 months with standard care, and 3-year PFS was 51.0% versus 26.5% (30). The unadjusted OS comparison was attenuated by substantial crossover, but crossover-adjusted analyses remained supportive of liso-cel. BELINDA, in contrast, did not improve EFS with tisa-cel; median EFS was 3.0 months in both groups (12). The median interval from randomization to infusion was approximately 52 days, and repeated bridging chemotherapy was common.

The discordance should not be reduced to a simplistic ranking of products. Differences in event definitions, bridging rules, manufacturing interval, crossover, disease burden at infusion, and the proportion of patients who actually received definitive therapy all influenced the observed results. Nevertheless, BELINDA also demonstrates that effective bridging cannot fully compensate for prolonged time to infusion in rapidly progressive disease. In aggressive LBCL, treatment velocity is a biological variable: delay permits clonal evolution, rising tumor burden, organ deterioration, corticosteroid exposure, and declining T-cell fitness. The second-line evidence therefore supports axi-cel or liso-cel for eligible patients with primary refractory or early relapsed disease, while preserving ASCT for selected patients with later, chemosensitive relapse (2, 3, 9–12, 30).

### 2.3 Long-term outcomes: durable remission, late relapse, and nonrelapse mortality

Long-term interpretation requires separating lymphoma control from overall survivorship. The flattening of PFS curves after approximately 2 years in pivotal studies suggests that some patients achieve operational cure (27–29). However, OS can continue to decline because of infection, persistent immune dysfunction, cardiovascular disease, subsequent malignancies, and other nonrelapse causes. In a 5-year standard-of-care axi-cel cohort, PFS and OS were 28.5% and 40.3%, respectively, while 5-year nonrelapse mortality reached 16.2%; more than half of nonrelapse deaths occurred beyond 2 years and were driven mainly by infection and subsequent malignancy (26).

This distinction changes the clinical endpoint of interest. Early-phase studies appropriately emphasized ORR and CR, but mature evaluation should include duration of CR, treatment-free survival, lymphoma-specific survival, nonrelapse mortality, immune reconstitution, health-related quality of life, and functional recovery. A durable radiographic remission is clinically incomplete if it is accompanied by recurrent infection, chronic cytopenia, neurocognitive impairment, or inability to resume independent living. Long-term follow-up should therefore be integrated into product evaluation and not treated as a separate survivorship issue.

### 2.4 Patient selection and the probability of reaching infusion

Patient selection should not be framed as a binary question of CAR T-cell eligibility. It is a multidimensional assessment of lymphoma biology, disease tempo, organ function, inflammatory and marrow reserve, neurologic vulnerability, infection risk, caregiver support, geography, and the probability of safely completing leukapheresis, bridging, lymphodepletion, and infusion. Primary refractory or early relapsed disease favors CAR T-cell therapy over a salvage-ASCT strategy when treatment can be delivered promptly (9–12). By contrast, ASCT remains reasonable for selected patients with late relapse and chemotherapy-sensitive disease (2, 3).

Chronological age alone should not exclude CAR T-cell therapy. Real-world series show meaningful activity in older adults, although vulnerability to delirium, deconditioning, infection, prolonged cytopenia, and late nonrelapse mortality may be greater (26, 31). Comprehensive geriatric assessment, baseline cognition, mobility, nutrition, social support, and competing mortality risk are therefore more informative than age alone. For patients not intended for transplantation, the phase 2 PILOT study demonstrated that second-line liso-cel could achieve an ORR of 80% and a CR rate of 54%, supporting cellular therapy in carefully selected patients who are older or have comorbidities but retain adequate functional reserve (32).

Disease kinetics often determine feasibility more strongly than static eligibility criteria. High lactate dehydrogenase, bulky or rapidly progressive disease, extranodal involvement, poor performance status, and high inflammatory burden are associated with inferior outcomes and greater toxicity (23, 33). Bridging therapy should therefore be selected with two objectives: prevent clinical deterioration before infusion and reduce tumor burden without causing irreversible marrow or T-cell injury. The most informative selection question is not simply “Can this patient receive CAR T cells?” but “Can this patient reach infusion with controlled disease, preserved organ function, and sufficient immune reserve to benefit?”.

### 2.5 Real-world evidence: generalizability, product selection, and methodological caution

Real-world evidence extends pivotal findings to patients who are older, more frail, have organ dysfunction, poorer performance status, central nervous system involvement, or disease trajectories that would have excluded them from registrational trials (31, 34–36). In the US Lymphoma CAR T Consortium, 43% of patients treated with standard-of-care axi-cel would not have met ZUMA-1 eligibility criteria, yet the ORR and CR rate were 82% and 64%, respectively (26, 34). At 5 years, outcomes remained broadly concordant with ZUMA-1, supporting external validity while also revealing late infection, secondary malignancy, and nonrelapse mortality as clinically important endpoints (26).

Large registry data also clarify the real-world performance of tisa-cel. In a CIBMTR cohort of 1,159 patients with diffuse large B-cell lymphoma or high-grade B-cell lymphoma, the ORR was 59.5% and the CR rate was 44.5%; 24-month PFS and OS were 28.4% and 43.6%, respectively. Grade 3 or higher CRS and neurotoxicity occurred in 6.0% and 7.4% (37). Outcomes were more favorable in patients with normal lactate dehydrogenase and disease control at infusion, reinforcing the importance of tumor kinetics and bridging response rather than product identity alone.

Real-world comparisons suggest an efficacy-toxicity trade-off among products. Propensity-matched analyses have reported higher response and disease-control rates with axi-cel than with tisa-cel, accompanied by more frequent ICANS (35). Such studies are clinically informative but remain vulnerable to residual confounding from center practice, calendar time, product availability, referral patterns, disease tempo, bridging choice, and physician selection. There is no randomized head-to-head comparison of commercial products. Product choice should therefore integrate urgency, neurotoxicity risk, comorbidity, expected manufacturing time, prior therapy, center experience, and patient preference rather than rely on unadjusted cross-study response rates.

### 2.6 Product biology, bridging, and sequencing with bispecific antibodies

Commercial CD19 CAR T-cell products are not interchangeable biological preparations (5, 7, 8, 18). Axi-cel uses CD28 costimulation and is characterized by rapid expansion and strong early effector activity, a profile associated with substantial antitumor activity but also a higher incidence of inflammatory and neurologic toxicity in several comparative cohorts (5, 18, 35). Tisa-cel and liso-cel use 4-1BB costimulation, with slower expansion and generally lower rates of severe acute toxicity (7, 8, 18). Liso-cel also uses a defined CD4-positive/CD8-positive composition. These biological differences are relevant, but they should be interpreted alongside the operational pathway because vein-to-vein time and disease control before infusion can outweigh modest product-level differences.

The development of CD20xCD3 bispecific antibodies has expanded the therapeutic landscape (38, 39). Glofitamab and epcoritamab offer off-the-shelf availability and can induce responses after CAR T-cell failure (38–40). CAR T-cell therapy and bispecific antibodies should be viewed as complementary immune-redirection platforms rather than direct substitutes. CAR T cells may be preferred when a one-time therapy with curative potential can be delivered safely; bispecific antibodies are particularly attractive when disease requires immediate treatment, manufacturing is not feasible, frailty limits cellular therapy, or relapse occurs after CAR T-cell infusion. Prospective sequencing studies are needed because retrospective comparisons are confounded by disease tempo and prior-treatment selection (40).

### 2.7 Combination strategies and next-generation platforms

Relapse after CAR T-cell therapy has encouraged rational combinations, but combinations should be linked to a defined resistance mechanism. Checkpoint blockade may restore function in selected patients with PD-1/PD-L1-dominant suppression, yet variable results indicate that it cannot reverse fixed epigenetic exhaustion, antigen loss, or profound myeloid suppression (41). Targeted agents, radiotherapy, and bispecific antibodies may be useful as bridging, consolidation, or salvage, but their timing and patient selection require prospective testing.

Next-generation engineering may be more transformative than empiric drug addiction. Dual-target CAR T cells directed against CD19/CD20 or CD19/CD22 aim to reduce antigen escape (42). Armored or logic-gated constructs seek to resist inhibitory signaling or remodel suppressive myeloid networks (13–15). Allogeneic CAR T-cell and CAR-NK platforms could shorten treatment timelines and broaden access (43), although host rejection, limited persistence, graft-versus-host disease, and gene-editing safety remain important barriers (43, 44). The clinical evidence and its major interpretive limitations are summarized in **Tables 1**, **2**.

**Table 1:** Clinical positioning and key efficacy outcomes of CAR-T cell products and related immune-redirection strategies in relapsed or refractory LBCL.

Platform/evidence base	Key biological or clinical features	Key efficacy outcomes from clinical evidence	Current role in LBCL	Major limitations or unresolved issues	Key references
Axi-cel; ZUMA-1 and ZUMA-7	CD19-directed autologous CAR-T with CD28 co-stimulation; rapid *in vivo* expansion and potent early effector activity.	ZUMA-1: ORR 83% and CR 58% among treated patients; 5-year PFS and OS approximately 32% and 43%, respectively. ZUMA-7: EFS 8.3 vs 2.0 months and CR 65% vs 32% versus standard care; 4-year OS 54.6% vs 46.0% (HR for death, 0.73).	Established activity in late-line R/R LBCL and preferred second-line option for selected patients with primary refractory disease or relapse within 12 months when treatment can be delivered promptly.	Higher inflammatory toxicity risk, including CRS and ICANS; outcomes remain affected by tumor burden, host inflammation, disease control at infusion, manufacturing interval, and CAR-T fitness.	(5, 9, 10, 18, 27)
Tisa-cel; JULIET and BELINDA	CD19-directed autologous CAR-T with 4-1BB co-stimulation; slower expansion and longer persistence compared with CD28-based products.	JULIET: ORR approximately 53% and CR 39%; 5-year PFS and OS approximately 28% and 32%, respectively; 60-month relapse-free probability 61% among responders. BELINDA: no EFS improvement; median EFS 3.0 months in both arms.	Active option in heavily pretreated R/R LBCL; randomized second-line BELINDA did not show superiority over standard care.	Clinical performance may be influenced by disease kinetics, bridging strategy, manufacturing interval, attrition before infusion, and trial design.	(7, 12, 18, 28)
Liso-cel; TRANSCEND NHL 001, TRANSFORM, and PILOT	CD19-directed autologous CAR-T with 4-1BB co-stimulation and defined CD4/CD8 composition; generally favorable tolerability profile.	TRANSCEND NHL 001: ORR 73% and CR 53%; extended follow-up median DOR, PFS, and OS were 23.1, 6.8, and 27.3 months, respectively. TRANSFORM: median EFS 29.5 vs 2.4 months and 3-year PFS 51.0% vs 26.5% versus standard care. PILOT: ORR 80% and CR 54% in selected transplant-ineligible second-line patients.	Effective in R/R LBCL and supported as second-line therapy for early relapsed or refractory disease; may be considered in carefully selected older or comorbid patients with adequate functional reserve.	Access, manufacturing time, and durability in biologically high-risk disease remain practical and clinical challenges.	(8, 11, 18, 29, 30, 32)
Salvage chemotherapy followed by ASCT	Historically standard approach for chemosensitive relapse; depends on disease control after platinum-based salvage therapy.	Comparator standard-care arms in second-line trials showed limited benefit in early refractory or relapsed disease, including median EFS of 2.0 months in ZUMA-7 and 2.4 months in TRANSFORM; BELINDA reported median EFS of 3.0 months in both arms.	Remains relevant for selected patients with late, chemosensitive relapse.	Limited benefit in primary refractory or early relapsed disease; many patients are ineligible because of age, comorbidity, poor response, organ dysfunction, or disease tempo.	(2–4, 9–12, 30)
CD20xCD3 bispecific antibodies; glofitamab and epcoritamab	Off-the-shelf T-cell redirection without individualized manufacturing; repeat dosing; outpatient potential.	Can induce responses in R/R LBCL, including after CAR-T failure, and provide rapid off-the-shelf immune redirection; randomized sequencing comparisons with commercial CAR-T products are still lacking.	Complementary to CAR-T, particularly when rapid access is needed, CAR-T is not feasible, frailty limits cellular therapy, or relapse occurs after CAR-T.	Optimal sequencing before or after CAR-T remains undefined; efficacy depends on endogenous T-cell reserve and retained CD20 expression.	(38–40)
Next-generation immune-cell platforms and combinations	Includes dual-target CAR-T, armored or logic-gated constructs, checkpoint blockade, allogeneic CAR-T/CAR-NK, and *in vivo* CAR programming.	Designed to improve durability by reducing antigen escape, enhancing T-cell fitness, remodeling suppressive microenvironments, or shortening manufacturing delay; most efficacy evidence remains early-phase or investigational.	Potential future strategies for patients with antigen escape, poor CAR-T fitness, suppressive tumor microenvironments, or urgent need for faster cellular therapy.	Most strategies require prospective validation; barriers include durability, host rejection, toxicity, graft-versus-host disease, gene-editing safety, and scalable delivery.	(41–44)

ASCT, autologous stem cell transplantation; CAR, chimeric antigen receptor; CR, complete response; CRS, cytokine release syndrome; DOR, duration of response; EFS, event-free survival; HR, hazard ratio; ICANS, immune effector cell-associated neurotoxicity syndrome; LBCL, large B-cell lymphoma; ORR, objective response rate; OS, overall survival; PFS, progression-free survival; R/R, relapsed or refractory.

**Table 2:** Mechanism-guided framework for resistance, toxicity, and biomarker-directed intervention after CD19 CAR-T therapy in LBCL.

Biological domain	Representative features/biomarkers	Clinical consequence	Assessment strategy	Mechanism-guided implication	Key references
Antigen escape	CD19 loss or downregulation, epitope alteration, alternative splicing, impaired surface trafficking, trogocytosis, or lineage plasticity.	CD19-negative or antigen-low relapse; reduced immune synapse formation and CAR-T activation.	Repeat biopsy with CD19 assessment and, where feasible, antigen-density evaluation.	Consider dual-target CAR-T or alternative antigen-directed strategies when relapse is driven by target loss.	(17, 18, 35)
Tumor-intrinsic resistance despite antigen retention	Preserved CD19 with altered tumor susceptibility, interferon-related signaling, impaired apoptosis, or resistant subclonal evolution.	Primary resistance or relapse despite detectable target antigen.	Biopsy-based genomics, transcriptomics, and tumor immune-contexture assessment.	Select salvage according to dominant biology rather than CD19 status alone; consider clinical trials or non-CD19 approaches when appropriate.	(14, 16, 18)
CAR-T cell fitness and exhaustion	Low stem-like memory features, terminal differentiation, poor proliferative reserve, metabolic insufficiency, and inhibitory receptors such as PD-1, TIM-3, TIGIT, and LAG-3.	Inadequate expansion, impaired cytotoxicity, early relapse, or failure of durable immune surveillance.	Infusion-product profiling, *in vivo* expansion kinetics, immune phenotyping, and longitudinal immune monitoring.	Optimize leukapheresis/manufacturing, reduce avoidable T-cell damage, and use rational combinations such as checkpoint blockade only in selected contexts.	(18, 19, 34)
Suppressive tumor microenvironment	Tumor-associated macrophages, myeloid-derived suppressor cells, regulatory T cells, stromal barriers, hypoxia, PD-L1 expression, IL-10, TGF-beta, IDO, and arginase activity.	Impaired trafficking, immune exclusion, reduced effector function, and relapse with retained antigen.	Pretreatment or relapse biopsy, single-cell profiling, spatial immune analysis, and PET-defined lesion heterogeneity.	Develop myeloid-directed, checkpoint-directed, radiotherapy-assisted, or armored CAR strategies for immune-excluded lesions.	(14–16)
Host inflammation and hematopoietic reserve	High LDH, CRP, ferritin, cytopenias, thrombocytopenia, neutropenia, and elevated metabolic tumor burden.	Inferior response, severe CRS/ICANS risk, prolonged cytopenias, infection, and hospitalization.	Routine laboratory risk stratification, CAR-HEMATOTOX, baseline PET-derived tumor burden, and early inflammatory surveillance.	Use risk-adapted bridging, inpatient/outpatient planning, early toxicity management, growth-factor support, and antimicrobial prophylaxis.	(20–24, 41)
Molecular and imaging response	Early ctDNA clearance or persistence; PET metabolic tumor volume, total lesion glycolysis, dissemination, radiomic texture, and immune-imaging signals.	Early molecular persistence may precede radiographic relapse; high tumor burden predicts resistance and toxicity.	Serial ctDNA monitoring, FDG-PET/CT response assessment, radiomics, and validated AI-based risk models.	Use adaptive surveillance and consider pre-emptive intervention for high-risk molecular persistence after prospective validation.	(39–43)
Immune reconstitution and survivorship	B-cell aplasia, hypogammaglobulinemia, delayed CD4-positive T-cell recovery, chronic cytopenias, recurrent infection, fatigue, and neurocognitive symptoms.	Late morbidity despite remission; impaired functional recovery and quality of survival.	Longitudinal immune monitoring, infection history, immunoglobulin levels, vaccination planning, and survivorship assessment.	Integrate prophylaxis, immunoglobulin replacement in selected patients, vaccination, neurocognitive evaluation, and long-term surveillance into CAR-T programs.	(25, 44)

AI, artificial intelligence; CAR, chimeric antigen receptor; CRS, cytokine release syndrome; ctDNA, circulating tumor DNA; FDG-PET/CT, fluorodeoxyglucose positron emission tomography/computed tomography; ICANS, immune effector cell-associated neurotoxicity syndrome; IDO, indoleamine 2,3-dioxygenase; LBCL, large B-cell lymphoma; TGF-beta, transforming growth factor-beta.

## 3 Mechanisms of resistance and relapse after CAR T-cell therapy

**Figure 1:**
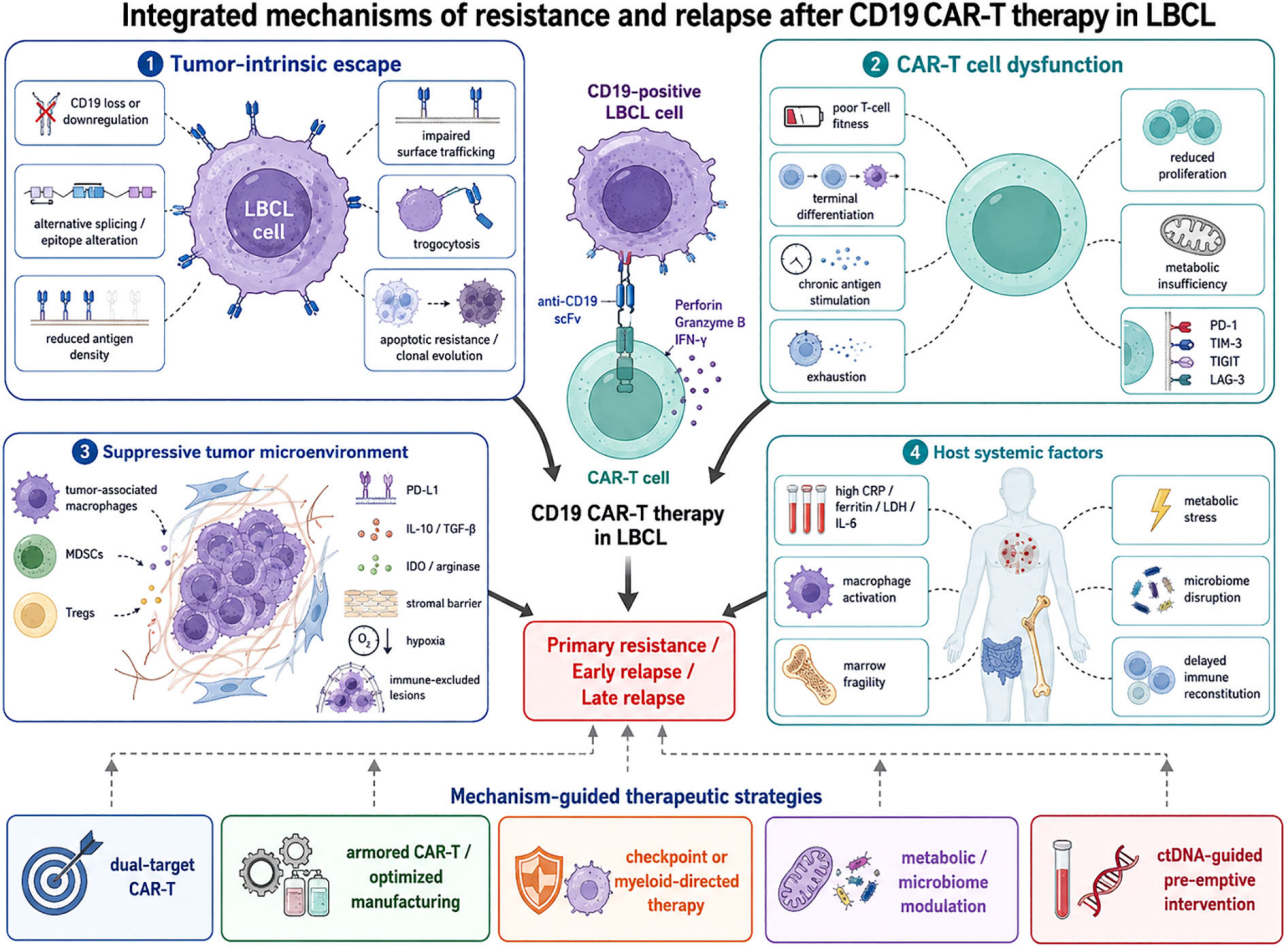
Integrated mechanisms of resistance and relapse after CD19 CAR-T therapy in LBCL. Tumor-intrinsic escape, CAR-T cell dysfunction, suppressive tumor microenvironmental programs, and host systemic factors jointly shape primary resistance, early relapse, and late relapse after CD19-directed CAR-T therapy. Mechanism-guided strategies include dual-target CAR-T, optimized manufacturing, checkpoint or myeloid-directed therapy, metabolic or microbiome modulation, and ctDNA-guided pre-emptive intervention.

Resistance and relapse after CD19-directed CAR-T therapy in LBCL should be understood as a dynamic, systems-level process rather than the consequence of a single dominant lesion (13–18). Although loss or downregulation of CD19 is the most intuitive mechanism of immune escape, many patients relapse despite retained target expression, indicating that antigen preservation alone is insufficient to guarantee CAR-T sensitivity (16, 17). Treatment failure emerges from the interaction among tumor-intrinsic evolution, CAR-T cell dysfunction, suppressive tumor microenvironments, systemic inflammation, metabolic stress, and inadequate endogenous immune cooperation (13–18). These mechanisms often coexist and reinforce one another, which explains why isolated interventions rarely restore durable disease control once resistance is established (**Figure 1**).

Antigen escape remains an important but incomplete explanation for relapse (16, 17). Under selective pressure from CD19-directed therapy, lymphoma cells may lose or reduce CD19 expression through genetic alteration, alternative splicing, epitope disruption, impaired surface trafficking, trogocytosis, or lineage plasticity (16, 17). Even partial reductions in antigen density can weaken immune synapse formation, increase the activation threshold required for CAR-T killing, and permit outgrowth of resistant subclones (16, 17). However, CD19-negative relapse accounts for only a subset of failures in LBCL (16, 17). Many recurrent tumors retain CD19 yet resist immune clearance, suggesting that target expression must be interpreted together with antigen density, spatial heterogeneity, downstream apoptotic competence, and the ability of CAR-T cells to traffic into and function within tumor sites (16, 17). This also explains why dual-antigen strategies targeting CD19/CD20 or CD19/CD22 may reduce single-antigen escape (42) but will not fully overcome resistance driven by impaired tumor susceptibility or hostile immune ecology.

CAR-T cell fitness is another central determinant of therapeutic durability (17, 18). Effective disease control requires not only initial expansion but also sustained proliferative capacity, cytotoxic function, metabolic adaptability, and resistance to exhaustion (17, 18). Patients with heavily pretreated LBCL often have autologous T cells compromised by prior chemotherapy, corticosteroid exposure, chronic inflammation, infection, and tumor-induced immune suppression (17, 18). These baseline defects may be further shaped during manufacturing, where excessive activation, prolonged culture, or unfavorable cellular composition can reduce stem-like memory features and promote terminal differentiation (18). After infusion, high tumor burden and persistent antigen stimulation can accelerate exhaustion, characterized by reduced cytokine production, impaired cytotoxicity, diminished proliferative reserve, metabolic insufficiency, and increased expression of inhibitory receptors such as PD-1, TIM-3, TIGIT, and LAG-3 (17, 18). Importantly, exhaustion is not merely a surface-marker phenotype but a transcriptional and epigenetic state that may become difficult to reverse. Future therapeutic strategies should therefore prioritize preservation of less differentiated T-cell states, optimization of manufacturing conditions, and biomarker-guided interventions that address specific forms of functional failure rather than empiric combination therapy.

The tumor microenvironment provides a critical anatomical and biological context for resistance (13–15). In LBCL, suppressive macrophages, myeloid-derived suppressor cells, regulatory T cells, stromal barriers, hypoxia, inhibitory cytokines, and checkpoint ligand expression can limit CAR-T trafficking, expansion, immune synapse formation, and effector function (13–15). Tumor-associated macrophages are particularly important because they may simultaneously amplify inflammatory toxicity and restrain antitumor immunity through IL-10, TGF-beta, prostaglandins, indoleamine 2,3-dioxygenase, arginase activity, and PD-L1 expression (13–15). Stromal remodeling and abnormal vasculature may further create immune-excluded lesions in which CAR-T cells fail to penetrate effectively despite adequate systemic expansion (13–15). These observations suggest that durable response may require cooperation between infused CAR-T cells and endogenous immunity (14, 15). Responding patients often show broader immune activation and favorable remodeling of the immune ecosystem, whereas resistant disease may reflect failure of endogenous antigen presentation, impaired recruitment of non-engineered T cells, and persistence of suppressive myeloid circuits (14, 15). CAR-T therapy should therefore be viewed not only as a direct cytotoxic product but also as a potential catalyst for immune reprogramming.

Host inflammatory and metabolic states further shape both resistance and relapse. Elevated baseline CRP, ferritin, LDH, IL-6, and related inflammatory markers are repeatedly associated with inferior response, greater toxicity, prolonged cytopenias, infection risk, and reduced survival (23). These markers likely capture overlapping biological processes, including aggressive tumor kinetics, macrophage activation, tissue injury, impaired hematopoietic reserve, and systemic immune dysregulation (23). Importantly, robust CAR-T expansion in a highly inflamed host may appear quantitatively adequate but remain functionally ineffective if the expanding cells are metabolically stressed or rapidly exhausted (17, 18). Metabolic constraints within the tumor microenvironment, including hypoxia, nutrient deprivation, adenosine accumulation, lactate enrichment, and oxidative stress, may further impair CAR-T proliferation and cytotoxicity while favoring suppressive myeloid and regulatory populations (13–15). The gut microbiome is also emerging as a modulator of systemic immune tone, treatment-related inflammation, and CAR-T efficacy (45). Antibiotic exposure, reduced microbial diversity, and altered microbiota-derived metabolites may influence T-cell activation, epithelial barrier integrity, and inflammatory set points (45). Although these findings require prospective validation before routine clinical implementation, they reinforce the concept that CAR-T outcome is influenced by host immune ecology as much as by the engineered product itself.

Relapse after CAR-T therapy reflects convergence among antigen modulation, impaired CAR-T fitness, tumor microenvironmental suppression, systemic inflammation, and host immune dysregulation (15–17). This framework has practical implications. Post-relapse evaluation should not be limited to CD19 testing alone, but should include assessment of antigen density, biopsy-based tumor biology, immune contexture, inflammatory state, and, where feasible, circulating tumor DNA or other dynamic disease markers (15–17, 46, 47). Similarly, next-generation therapeutic strategies should be matched to dominant resistance biology: dual-target CAR-T cells for antigen escape (42), manufacturing or engineering approaches for poor T-cell fitness (18), checkpoint or myeloid-directed combinations for immune suppression (13–15, 41), metabolic or microbiome-preserving strategies for host immune dysfunction (45), and pre-emptive intervention for patients with early molecular persistence (46, 47). The field should move from descriptive relapse classification toward mechanism-guided salvage and prevention, because durable improvement will depend on identifying not only whether CAR-T therapy fails, but why it fails in each patient.

## 4 Toxicities and their biological basis

Toxicity after CD19-directed CAR-T cell therapy in LBCL is not a nonspecific consequence of immune activation, but rather reflects a coordinated interaction among CAR-T expansion, tumor burden, host inflammatory tone, endothelial integrity, myeloid-cell activation, and delayed immune reconstitution (19–24). Although acute immune-mediated toxicities such as cytokine release syndrome (CRS) and immune effector cell-associated neurotoxicity syndrome (ICANS) dominate early clinical management (19–22), later complications—including prolonged cytopenias, infections, hypogammaglobulinemia, and impaired functional recovery—are equally important determinants of morbidity, treatment feasibility, and long-term survivorship (23–25). Understanding these toxicities as biologically linked rather than isolated events is essential for risk-adapted patient selection, early intervention, and safer therapeutic engineering.

CRS is the prototypical inflammatory toxicity of CAR-T therapy (19). It is initiated by CAR engagement and T-cell activation but is amplified predominantly through host monocytes and macrophages (20, 21). Activated CAR-T cells release interferon-gamma, TNF-alpha, GM-CSF, and other mediators that stimulate myeloid cells to produce IL-6, IL-1, and additional inflammatory cytokines, leading to fever, hypotension, hypoxia, capillary leak, coagulopathy, and, in severe cases, multiorgan dysfunction (19–21). The risk and severity of CRS are influenced by baseline tumor burden, systemic inflammation, product-specific expansion kinetics, and early *in vivo* CAR-T proliferation (19–22). CD28-costimulated products, which typically expand rapidly, tend to produce more intense early inflammatory syndromes (5, 18), whereas 4-1BB-based products are generally associated with slower expansion and lower rates of severe acute toxicity (7, 8, 18). The clinical introduction of tocilizumab has transformed CRS management by interrupting IL-6 receptor signaling, and corticosteroids are increasingly used earlier in severe or refractory cases (19–21). Importantly, appropriately timed toxicity-directed immunosuppression does not appear to universally compromise efficacy, although the timing, cumulative dose, and biological context of immunosuppression remain relevant.

ICANS overlaps temporally with CRS but is biologically distinct (19, 22). Its pathogenesis involves endothelial activation, blood-brain barrier disruption, cytokine-mediated neuroinflammation, coagulation abnormalities, and trafficking of immune cells or soluble inflammatory mediators into the central nervous system (22). Clinically, ICANS may manifest as handwriting deterioration, aphasia, tremor, confusion, encephalopathy, seizures, or, rarely, cerebral edema (19). Unlike CRS, isolated ICANS is less responsive to IL-6 receptor blockade, likely because tocilizumab does not adequately suppress central nervous system inflammation and may increase circulating IL-6 levels (19, 22). Corticosteroids therefore remain the mainstay of treatment for clinically significant neurotoxicity, while IL-1 blockade, endothelial-protective strategies, and earlier biomarker-driven intervention are under investigation (19, 22). The biology of ICANS suggests that neurotoxicity is not determined solely by CAR-T expansion, but by the interaction between inflammatory intensity, vascular vulnerability, coagulation disturbance, and host neurologic reserve.

Beyond acute inflammatory syndromes, hematologic and infectious toxicities represent a major and sometimes underappreciated burden after CAR-T therapy (23, 24). Prolonged neutropenia, thrombocytopenia, and anemia may persist for weeks or months after infusion and arise from overlapping mechanisms, including prior therapy-related marrow injury, lymphodepleting chemotherapy, baseline inflammatory activation, cytokine-mediated suppression of hematopoietic stem and progenitor cells, immune-mediated marrow dysfunction, infection-related stress, and disease involvement of the marrow (23). These cytopenias are clinically important because they increase the risk of infection, bleeding, transfusion dependence, hospitalization, and treatment delay (23). Composite tools such as CAR-HEMATOTOX (23) are useful because they integrate inflammatory and hematopoietic reserve parameters and may help identify patients who require intensified monitoring, early growth-factor support, or individualized antimicrobial prophylaxis.

Infections after CAR-T therapy reflect a continuum of immune vulnerability (24). Early infections are often driven by neutropenia, mucosal injury, hospitalization, and intensive supportive care, whereas later infections are more closely related to delayed immune reconstitution, B-cell aplasia, hypogammaglobulinemia, impaired CD4-positive T-cell recovery, corticosteroid exposure, and repeated antimicrobial pressure (24). Risk-adapted antibacterial, antiviral, and antifungal prophylaxis, immunoglobulin replacement in selected patients, vaccination planning, and longitudinal immune monitoring should therefore be considered integral components of CAR-T delivery rather than ancillary supportive measures (24). As CAR-T therapy moves earlier in the treatment course and is offered to older or more medically complex patients, reducing infectious morbidity will become increasingly important for expanding safe access.

Long-term survivorship after CAR-T therapy is now an emerging clinical priority (25). Durable remission does not necessarily equate to full biological or functional recovery (25). Patients may experience persistent fatigue, neurocognitive symptoms, chronic cytopenias, recurrent infections, hypogammaglobulinemia, endocrine dysfunction, psychosocial stress, and financial toxicity (24, 25). Secondary malignancies appear uncommon but require systematic surveillance, particularly as follow-up lengthens and cellular therapy is used earlier in LBCL (25). A mature CAR-T program should therefore include not only acute toxicity algorithms but also structured survivorship care, including immune reconstitution monitoring, infection prevention, neurologic and cognitive assessment when clinically indicated, quality-of-life evaluation, and coordination with primary care (24, 25). In this sense, the next benchmark for CAR-T safety should extend beyond reducing grade 3 or higher CRS and ICANS; it should also include preservation of immune resilience, functional recovery, and long-term quality of survival.

## 5 Biomarkers and precision CAR T-cell therapy

**Figure 2:**
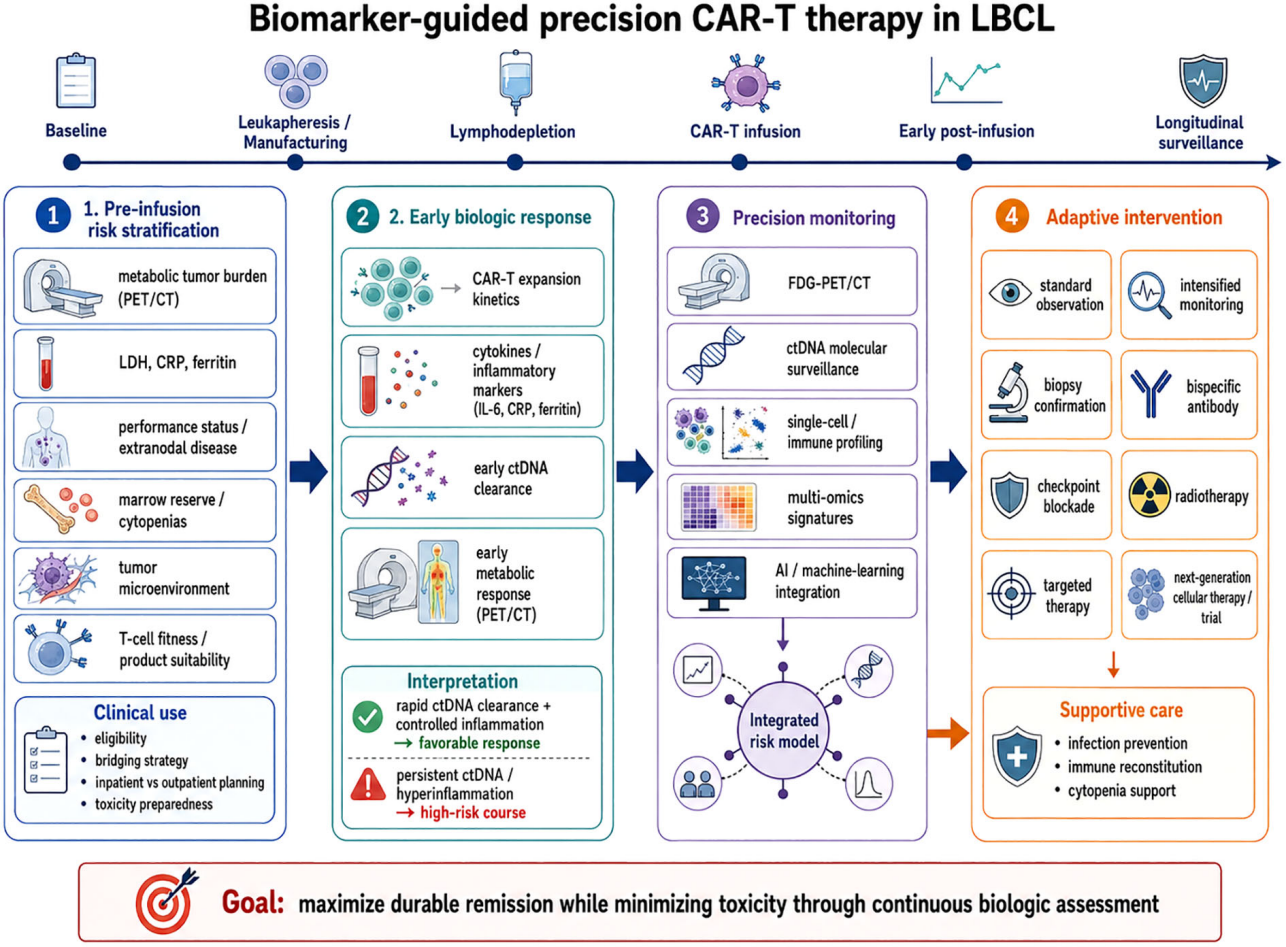
Biomarker-guided precision CAR-T therapy in LBCL. Precision CAR-T therapy requires continuous biologic assessment across baseline risk stratification, leukapheresis/manufacturing, lymphodepletion, infusion, early post-infusion response, and longitudinal surveillance. Integrated biomarkers including PET/CT tumor burden, inflammatory markers, CAR-T expansion kinetics, ctDNA clearance, immune profiling, multi-omics, and AI-based models may support adaptive intervention and toxicity minimization.

Precision CAR-T therapy in LBCL requires a transition from empiric treatment delivery to biology-informed decision-making (15, 18, 46, 47). Current outcomes are shaped by tumor burden, tumor-intrinsic immune escape, CAR-T product fitness, host inflammatory state, marrow reserve, tumor microenvironmental architecture, and post-infusion immune recovery (15, 18, 23). No single biomarker can capture this complexity. The most clinically useful strategy is therefore likely to involve integrated models that combine baseline clinical variables, dynamic molecular response, metabolic imaging, immune profiling, and computational prediction (33, 46–49). Such models should not merely estimate prognosis; they should guide practical decisions regarding patient selection, bridging therapy, product choice, toxicity prevention, post-infusion monitoring, and pre-emptive intervention (**Figure 2**).

Clinical and laboratory biomarkers remain the foundation of risk assessment because they are widely available and directly actionable. High metabolic tumor burden, elevated LDH, extranodal disease, poor performance status, thrombocytopenia, neutropenia, elevated CRP, ferritin, and impaired marrow reserve are consistently associated with inferior outcomes or increased toxicity (23, 33). These variables should not be interpreted as simple statistical predictors; biologically, they reflect aggressive disease kinetics, systemic inflammation, hematopoietic fragility, and reduced immune competence (23, 33). Composite scores such as CAR-HEMATOTOX illustrate how routine clinical data can be converted into risk-adapted supportive care strategies (23). Similarly, PET-derived measures such as metabolic tumor volume and lesion dissemination provide information beyond conventional staging by capturing disease bulk, spatial distribution, and inflammatory burden (33, 48). In the near term, these biomarkers are most useful for trial stratification, bridging therapy selection, inpatient versus outpatient planning, early toxicity surveillance, and individualized supportive care.

Circulating tumor DNA is one of the most promising tools for dynamic disease monitoring after CAR-T therapy (46, 47). Although FDG-PET/CT remains indispensable, imaging can be confounded by inflammation, pseudoprogression, delayed metabolic resolution, and treatment-related tissue changes (48). ctDNA provides a molecular measure of residual tumor burden and may identify impending treatment failure before overt radiographic progression (46, 47). Rapid ctDNA clearance within the first weeks after infusion is associated with favorable outcomes, whereas persistent or rising ctDNA suggests molecular resistance and a high risk of relapse (46, 47). This creates a rational framework for response-adapted intervention, in which patients with early molecular persistence could be considered for intensified surveillance, biopsy confirmation, bispecific antibodies, checkpoint blockade, radiotherapy, targeted agents, or investigational cellular approaches before clinically aggressive relapse becomes established (46, 47). To become routine, ctDNA assays will require standardization of timing, sensitivity, lymphoma-specific mutation tracking, and clinically validated intervention thresholds.

Imaging biomarkers are also evolving beyond conventional response assessment. Baseline FDG-PET/CT can quantify metabolic tumor volume, total lesion glycolysis, dissemination patterns, and lesion heterogeneity, all of which may correlate with both resistance and inflammatory toxicity (33, 49). Radiomic models may further extract spatial and textural features that are not captured by Lugano response criteria (48, 49). In parallel, functional immune imaging represents an emerging frontier. Tracers capable of visualizing CD8-positive T-cell infiltration, immune activation, or target antigen expression could help distinguish immune-permissive from immune-excluded disease sites and may eventually guide localized radiotherapy, combination immunotherapy, or selection of alternative cellular platforms (49). However, radiomics and immune imaging must undergo prospective validation, harmonization across scanners and reconstruction protocols, and demonstration of clinical utility before they can guide routine CAR-T decisions.

High-dimensional immune and molecular profiling provides a deeper view of why CAR-T therapy succeeds or fails (13–18). Single-cell sequencing can characterize the infusion product, endogenous immune compartment, tumor cells, and suppressive myeloid populations with a resolution that bulk assays cannot provide (13, 14, 18). Durable response has been associated with less differentiated, proliferative, and stem-like memory T-cell states, whereas resistance may involve exhausted CAR-T phenotypes, dysfunctional endogenous T cells, suppressive macrophage programs, antigen modulation, impaired apoptosis, and inflammatory or metabolic stress signatures (13–18). Multi-omics approaches—including genomics, transcriptomics, epigenomics, proteomics, metabolomics, and microbiome profiling—can link tumor-intrinsic risk with host immune ecology (17, 45). The clinical challenge is not to generate more data, but to define reproducible signatures that are interpretable, scalable, and actionable. For example, a useful multi-omics model should be able to identify whether a patient is most likely to fail because of antigen escape, poor CAR-T fitness, myeloid suppression, high inflammatory burden, or insufficient immune reconstitution, and then match that biology to a rational intervention.

Artificial intelligence and machine-learning approaches may help integrate these multidimensional data streams into clinically usable prediction models (49). Potential applications include relapse prediction, toxicity forecasting, manufacturing failure risk, bridging therapy selection, image-based prognostication, and early identification of patients who require pre-emptive post-CAR-T therapy (49). The appeal of these models lies in their ability to detect nonlinear interactions among variables that conventional analyses may miss. However, predictive performance alone is insufficient. Models must be externally validated, calibrated across institutions, interpretable to clinicians, and tested prospectively for whether their use improves patient outcomes (49). Algorithms trained on single-center referral patterns, product distributions, imaging protocols, or supportive care practices may not generalize to broader populations. Therefore, the future of AI in CAR-T therapy should prioritize clinical utility, transparency, and decision impact rather than accuracy metrics alone.

Ultimately, biomarker development should move CAR-T therapy toward an adaptive therapeutic model (15, 46, 47). Before infusion, integrated biomarkers could inform eligibility, product selection, bridging strategy, toxicity preparedness, and whether alternative immune-redirection approaches may be more appropriate (15, 18, 23). During the early post-infusion phase, inflammatory markers, CAR-T expansion kinetics, ctDNA clearance, and imaging response could identify patients who are likely cured versus those with emerging resistance (46, 47). During survivorship, immune reconstitution, hypogammaglobulinemia, cytopenia recovery, infection history, and molecular surveillance could guide supportive care and relapse prevention (23–25). In this framework, precision CAR-T therapy is not defined by a single diagnostic test, but by continuous biological assessment across the treatment trajectory. The goal is to intervene before resistance becomes clinically irreversible while minimizing unnecessary toxicity in patients already destined for durable remission.

## 6 Discussion and future perspectives

The revised clinical evidence demonstrates that CAR T-cell therapy in LBCL is neither a single homogeneous intervention nor a treatment whose effectiveness can be summarized by response rate alone. Pivotal third-line studies establish durable remission in a minority of heavily pretreated patients, and randomized second-line trials show that earlier axi-cel or liso-cel can outperform a salvage-ASCT strategy in primary refractory or early relapsed disease (5–12, 27–30). At the same time, the divergence of ZUMA-7, TRANSFORM, and BELINDA shows that manufacturing interval, bridging strategy, analytic denominator, and disease progression before infusion are integral to efficacy rather than logistical footnotes.

Long-term data also refine the meaning of cure. A plateau in lymphoma progression after 2 years supports durable disease control, but late infection, immune dysfunction, cytopenia, and subsequent malignancy contribute to nonrelapse mortality and reduce OS (25, 26). Thus, the next benchmark should combine lymphoma-specific control with immune recovery, functional independence, quality of life, and avoidance of late treatment-related mortality. This is especially important as CAR T-cell therapy moves earlier and reaches older or medically complex patients.

A central biological lesson is that CAR T-cell outcome is not determined by a single variable (13–18, 45). CD19 density, tumor burden, apoptotic competence, co-stimulatory signaling, T-cell differentiation state, suppressive myeloid programs, vascular integrity, metabolic stress, microbiome composition, manufacturing time, and supportive care interact. This complexity creates multiple points for intervention but argues against empiric combination therapy. The dominant mechanism of failure should guide whether the next step is dual targeting, improved manufacturing, myeloid or checkpoint modulation, localized radiotherapy, a bispecific antibody, or pre-emptive treatment triggered by molecular persistence.

The next generation of CAR T-cell therapy will likely evolve along four axes. First, product engineering will incorporate dual-target constructs, armored CARs, logic-gated systems, and gene-edited allogeneic cells (42–44). Second, delivery will become faster through decentralized or point-of-care manufacturing and potentially *in vivo* CAR programming (44, 50). Third, patient selection will integrate clinical risk, metabolic tumor burden, inflammatory reserve, ctDNA, and immune profiling (23, 33, 46–49). Fourth, post-infusion management will become adaptive, using early molecular response and immune recovery to distinguish patients likely to achieve durable remission from those who need mechanism-matched intervention.

Allogeneic and off-the-shelf approaches could reduce manufacturing delay and expand access, but they must demonstrate persistence, efficacy, immune compatibility, and gene-editing safety comparable to autologous products (43). *In vivo* CAR programming is potentially more disruptive, yet tissue targeting, reversibility, control of transgene expression, and management of unexpected immune activation will be decisive (44). Across these innovations, access and health-system capacity remain as important as biological performance.

## 7 Conclusions

CD19 CAR T-cell therapy has established a curative treatment pathway for a subset of patients with LBCL, particularly when delivered promptly in appropriately selected patients. The clinical evidence is strongest when trial design, treatment feasibility, and long-term survivorship are interpreted together. Further progress will require rapid delivery, rigorous patient selection, biologically informed prevention of resistance, toxicity-aware survivorship, and prospective validation of biomarker-guided intervention. The objective is not merely a higher initial response rate, but durable, safe, and equitable cure.
